# GeneMANIA: Fast gene network construction and function prediction for Cytoscape

**DOI:** 10.12688/f1000research.4572.1

**Published:** 2014-07-01

**Authors:** Jason Montojo, Khalid Zuberi, Harold Rodriguez, Gary D. Bader, Quaid Morris

**Affiliations:** 1 Departments of Molecular Genetics and Computer Science, The Donnelly Centre, University of Toronto, Toronto, ON, M5S 3E1, Canada

## Abstract

The GeneMANIA Cytoscape app enables users to construct a composite gene-gene functional interaction network from a gene list. The resulting network includes the genes most related to the original list, and functional annotations from Gene Ontology. The edges are annotated with details about the publication or data source the interactions were derived from. The app leverages GeneMANIA’s database of 1800+ networks, containing over 500 million interactions spanning 8 organisms: *A. thaliana, C. elegans, D. melanogaster, D. rerio, H. sapiens, M. musculus, R. norvegicus*, and *S. cerevisiae*. Users may also import their own organisms, networks, and expression profiles. The app is compatible with Cytoscape versions 2 and 3.

## Introduction

The GeneMANIA Cytoscape ^1^ app enables users to construct a weighted composite functional interaction network from a list of genes. Each node represents a gene and its products. The app uses the GeneMANIA algorithm ^2^ to find other genes and gene products that are most related to the original list, and shows how they are related.

The app provides access to most of the features of the GeneMANIA prediction server ^3^ while removing limitations on gene list length, and the maximum size of the resulting network. The app also allows predictions to be made on user-defined organisms and arbitrarily large custom networks.

### Source networks

GeneMANIA uses a database of organism-specific weighted networks to construct the resulting composite network. The database includes over 1800 networks, containing over 500 million interactions for 8 organisms: *A. thaliana*, *C. elegans*, *D. melanogaster*, *D. rerio*, *H. sapiens*, *M. musculus*, *R. norvegicus*, and *S. cerevisiae*. The networks are organized into groups such as *co-expression*, where edges are derived from expression profiles, and *shared protein domains*, where edges represent genes that encode proteins with similar domains. Users may select any combination of these as the basis of the composite network they construct for their gene list.

### Gene scores

Prior to construction, the selected networks are each assigned a weight by the GeneMANIA algorithm. The weight of each edge is multiplied by the weight of the containing network. Next, the union of all edges in the network is taken. In the case of multiple edges between any pair of nodes, the edges are collapsed into one and assigned a weight equal to the sum of the individual edge weights. The query genes are assigned a label value of 1, while all other genes are 0. Label propagation is then applied to the entire network ^2^ and the resulting labels are saved as the score attribute in the node table. This score indicates the relevance of each gene to the original list based on the selected networks. Higher scores indicate genes that are more likely to be functionally related. Users may extend their original gene list by adding these top ranking genes to their network. They can also choose not to add any other genes so they can visualize how the members of their list are connected.

### Composite network

**Figure 1.**
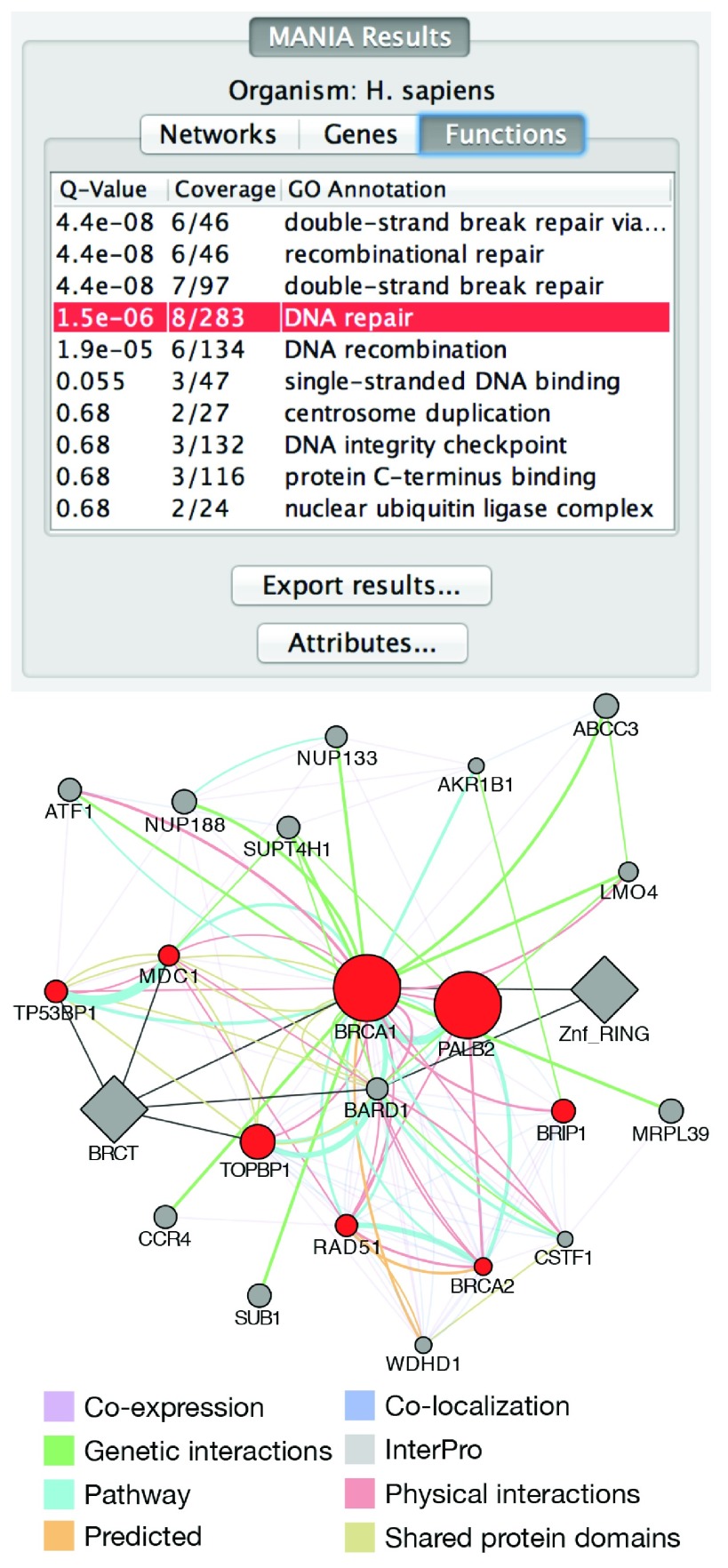
Composite network for *BRCA1*. The circles are genes and the diamonds are protein domain attributes. Up to 20 most related genes and 20 most related attributes are shown. The red genes are annotated with *DNA repair*, as indicated in the *Functions* tab.

**Figure 2.**
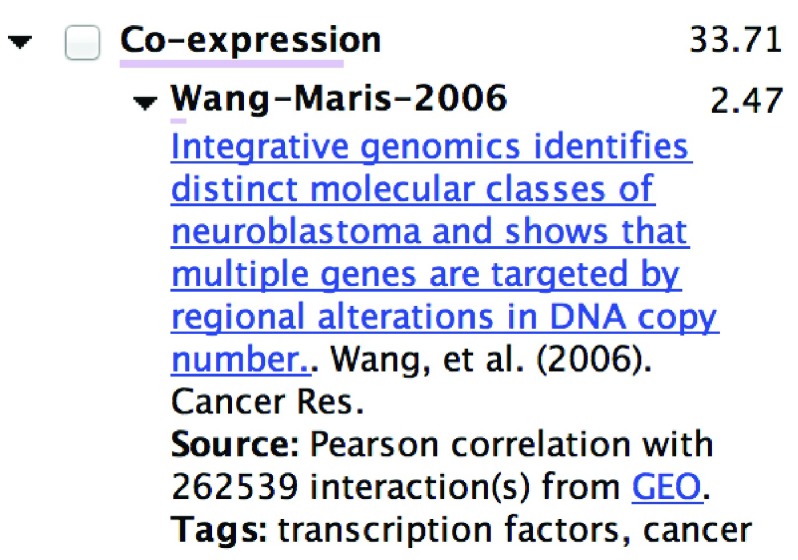
Sample of provenance details provided for each edge in composite network. The source networks are grouped by type (e.g. *co-expression*) and list each network weight, as well as the sum of the weights of the networks in each group. Citations and links to relevant publications and data sources and provided where possible.

Instead of providing the user with the composite network used during label propagation, the Cytoscape app displays at most one edge for each type of network that contributed to the gene scores ( Figure 1). For example, if five *co-expression* networks and two *physical interaction* networks contained an edge between the same pair of genes, the resulting network would contain one *co-expression* edge and one *physical interaction* edge for that pair. The edges are annotated with the original edge weights, the source networks from which those weights originate, relevant publications, and details about how the data was collected or processed ( Figure 2). The nodes are annotated with Gene Ontology ^4^ terms, alternate identifiers and synonyms.

## Implementation

**Figure 3.**
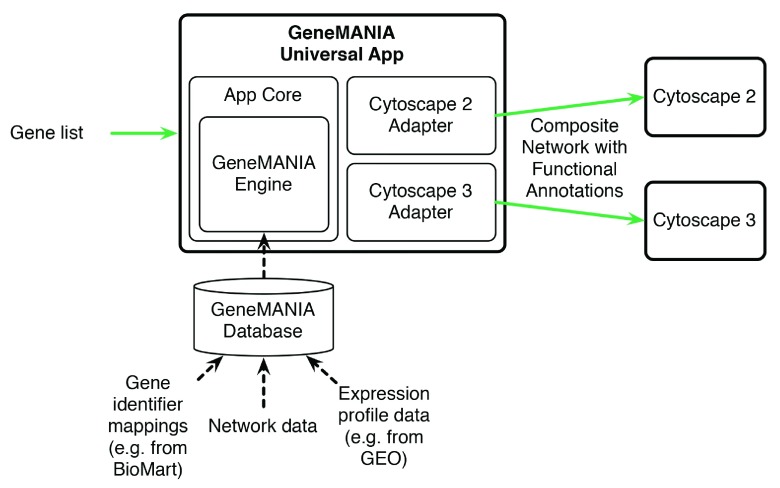
Architecture diagram of the GeneMANIA app illustrating the inputs and outputs of the system. The user-provided gene list is used to select the most relevant interactions from the GeneMANIA database. The resulting network is visualized in Cytoscape.

The GeneMANIA app is an update to the GeneMANIA plugin for Cytoscape 2 ^5^. The app preserves runtime compatibility with older versions of Cytoscape. It is distributed as a universal binary that runs on every release of Cytoscape since version 2.6.3. Figure 3 illustrates how we architected the software to enable the same code to run in multiple environments. The *GeneMANIA Engine* module, which implements the algorithm, is an independent layer that is also used directly by the GeneMANIA prediction web server. The *App Core* module includes highly parallelized command line tools for function prediction and cross validation ^6^ on multiprocessor clusters and multicore workstations. It also contains an abstraction layer to provide access to a small subset of Cytoscape’s functionality through high-level Application Programming Interface (API). This alternative API effectively decouples the app implementation from a particular version of Cytoscape, allowing the same code to drive a Cytoscape 2 plugin and Cytoscape 3 app.

### Database

The app provides access to all previous editions of the GeneMANIA database dating back to the initial *September 23, 2010* release. New data updates will also be supported as they become available. As of the *March 3, 2011* database release, two subsets of the data are available for users with special requirements. The *core* subset is roughly 20% of the size of the full database and only includes networks that are selected by default ^3^. The *open license* subset only includes network data with no restrictions on use. For example, networks derived from I2D ^7^ and HPRD ^8^ are excluded from this subset since their standard licenses prohibit commercial use of their data.

The networks are stored on disk as compact binary sparse matricies, which are used directly by GeneMANIA’s network integrator. This representation allows networks to be loaded quickly and used immediately without transformation into a different data structure. Gene and network metadata, including descriptions and provenance details, are stored in a Lucene index. This allows fast retrieval of metadata and gene name autocompletion as users type in their list.

### User-defined organisms and networks

Unlike the GeneMANIA prediction server which only supports 8 organisms, the app allows users to perform predictions on their own organisms. To import an organism into a user’s local database, the user needs to provide a tab-delimited file containing the organism’s genome, where each row contains the primary identifier of a gene followed by alternate identifiers and synonyms. From there, users may import tab-delimited network data or expression profiles. Users may also import networks or expression profiles they have loaded into Cytoscape. The app can also be used with non-biological data such as social networks, where the nodes are individuals and edges represent various relationships between them.

## Results

**Figure 4.**
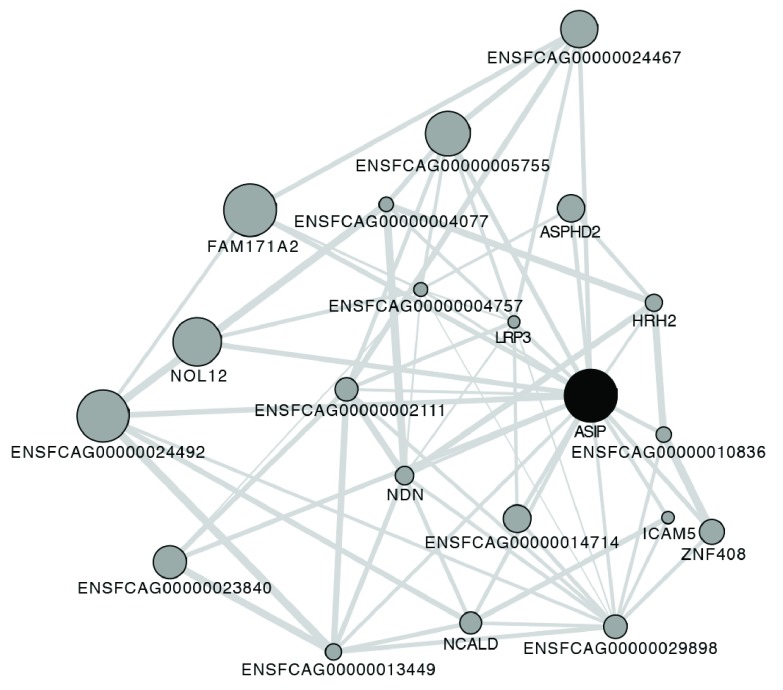
The 20 genes most related to *Felis catus* gene *ASIP*, based on GEO dataset GSE46431. The expression profiles from this dataset were converted into a co-expression network using the GeneMANIA app.

To demonstrate the steps involved with performing predictions on custom organisms not already provided by GeneMANIA, Ensembl ^9^ Gene IDs and their associated gene names for *Felis catus* were imported from BioMart ^10^ and imported into GeneMANIA as an organism. Data set GSE46431 was downloaded from the Gene Expression Omnibus (GEO) ^11^ and imported directly as expression profile data to yield a coexpression network. On a 2.3 GHz Intel Core i7 3615QM system with 16 GB RAM and SSD storage, it took approximately 5 minutes to import the data. Using this network, the app was used to find and display the 20 genes most related to ASIP, which took 1 second ( Figure 4).

## Conclusions

The GeneMANIA app extends the capabilities of the GeneMANIA prediction server by allowing users to quickly construct networks from gene lists for custom organisms and network data without imposing any limits on the size of the inputs or output while retaining provenance of the source data. The app also allows users to replicate past results by providing access to all publicly-released GeneMANIA datasets.

## Software availability

Software available from the Cytoscape’s App Manager or the App Store: http://apps.cytoscape.org/apps/GeneMania.

Latest source code: https://github.com/GeneMANIA/genemania.

Source code as at the time of publication: https://github.com/F1000Research/genemania/releases/tag/V1.0

Archived source code as at the time of publication: http://www.dx.doi.org/10.5281/zenodo.10523 ^12^

License: LGPL 2.1: https://www.gnu.org/licenses/old-licenses/lgpl-2.1.html
