# Supplementary material for: GeneMANIA: Fast gene network construction and function prediction for Cytoscape
Source: F1000Res. 2014 Jul 1;3:153. [Version 1] doi: 10.12688/f1000research.4572.1 (PMC4168749; doi:10.12688/f1000research.4572.1)
Supplement: Peer review history

# Peer review: Referee response for version 1

**DOI:** 10.5256/f1000research.4891.r5567

**Published:** 2014-08-01

**Authors:** Rosalba Giugno

**Affiliations:** 1 Department of Molecular Virology, Immunology and Medical Genetics, The Ohio State University, Columbus, OH, USA

The authors present an updated version of an affirmed work in the literature. A few comments follow.

The authors should highlight the upgrading aspects with respect to the previous work. Moreover, it would be useful to report the used database sources, the biggest retrieved network and how they deal with such a network with respect to the ‘unlimited’ list of genes (visualization and running time).

I have read this submission. I believe that I have an appropriate level of expertise to confirm that it is of an acceptable scientific standard.

# Peer review: Referee response for version 1

**DOI:** 10.5256/f1000research.4891.r5285

**Published:** 2014-07-21

**Authors:** Giovanni Scardoni

**Affiliations:** 1 Center for Biomedical Computing, University of Verona, Verona, Italy

In this paper the authors present the GeneMANIA Cytoscape app. It allows users to construct gene networks from a gene list. The paper is well written and complete.

**Minor comment:**

The database contains over 500 million interactions spanning 8 organisms: *A. thaliana, C. elegans, D. melanogaster, D. rerio, H. sapiens, M. musculus, R. norvegicus*, and *S. cerevisiae. *It is not clear how this interactions are obtained (literature, other database ...) More details about it would improve the paper.

I have read this submission. I believe that I have an appropriate level of expertise to confirm that it is of an acceptable scientific standard.
